# SensorHub: Multimodal Sensing in Real-Life Enables Home-Based Studies

**DOI:** 10.3390/s22010408

**Published:** 2022-01-05

**Authors:** Jonas Chromik, Kristina Kirsten, Arne Herdick, Arpita Mallikarjuna Kappattanavar, Bert Arnrich

**Affiliations:** Hasso Plattner Institute, University of Potsdam, 14482 Potsdam, Germany; arne.herdick@student.hpi.de (A.H.); arpita.kappattanavar@hpi.de (A.M.K.); bert.arnrich@hpi.de (B.A.)

**Keywords:** multimodal sensing, home-based studies, activity recognition, sensor systems, ecological momentary assessment, digital health

## Abstract

Observational studies are an important tool for determining whether the findings from controlled experiments can be transferred into scenarios that are closer to subjects’ real-life circumstances. A rigorous approach to observational studies involves collecting data from different sensors to comprehensively capture the situation of the subject. However, this leads to technical difficulties especially if the sensors are from different manufacturers, as multiple data collection tools have to run simultaneously. We present SensorHub, a system that can collect data from various wearable devices from different manufacturers, such as inertial measurement units, portable electrocardiographs, portable electroencephalographs, portable photoplethysmographs, and sensors for electrodermal activity. Additionally, our tool offers the possibility to include ecological momentary assessments (EMAs) in studies. Hence, SensorHub enables multimodal sensor data collection under real-world conditions and allows direct user feedback to be collected through questionnaires, enabling studies at home. In a first study with 11 participants, we successfully used SensorHub to record multiple signals with different devices and collected additional information with the help of EMAs. In addition, we evaluated SensorHub’s technical capabilities in several trials with up to 21 participants recording simultaneously using multiple sensors with sampling frequencies as high as 1000 Hz. We could show that although there is a theoretical limitation to the transmissible data rate, in practice this limitation is not an issue and data loss is rare. We conclude that with modern communication protocols and with the increasingly powerful smartphones and wearables, a system like our SensorHub establishes an interoperability framework to adequately combine consumer-grade sensing hardware which enables observational studies in real life.

## 1. Introduction

Clinical research is and remains one of the most important components for improving the general healthcare system. The two major components are controlled experiments and observational studies [1]. Controlled experiments aim at controlling all relevant variables and modulating only one variable at a time. This is usually achieved by a sanitized laboratory environment that creates a situation that is far from the subjects’ real-life conditions. Observational studies on the other hand try to observe people without removing possible influencing factors. Hence, observational studies are more prone to confounding factors but nevertheless important in order to, firstly, discover research questions and hypotheses that can then be studied rigorously through controlled experiments (as is the case with grounded theory research [2]), and secondly, to validate that findings from controlled experiments are applicable in real-world scenarios.

Due to advancements in wearable sensor technology, noninvasive studies can now also be carried out outside a laboratory hence enabling observational studies “in the wild”. However, collecting data from multiple devices from different manufacturers poses technical difficulties. Therefore, many studies are limited to unimodal data collection using only one type of device from one manufacturer for reasons of user convenience or of technical feasibility. This vastly limits the possibilities of sensor-based observational studies and hence this issue needs to be addressed. We present our system, called SensorHub, which enables multimodal sensing in real-life scenarios without overbearing the user with technical difficulties.

### Motivation and Problem Statement

Controlled experiments are an invaluable tool for determining the precise effect of a well-defined intervention under controlled conditions. However, they also suffer from shortcomings such as the high cost for the researchers, high effort for the subjects, non-natural setups which can influence the results, and vulnerability to external circumstances (e.g., outbreak of a pandemic). Therefore, controlled experiments need to be supplemented by observational studies in a setting that is closer to the real-life conditions of the subject. These studies, ideally, take place at home or in an environment that is familiar to the subject.

A major advantage of conducting studies in a non-laboratory setting is that non-laboratory studies lead to more realistic results because the subject does not feel observed. The effect that people behave differently when they are taking part in a study or being observed is known as the Hawthorne effect and was first described in the 1950s [3]. By now, the existence of the Hawthorne effect could be validated by a randomized, controlled trial [4]. The Hawthorne effect refers to subjects changing their behavior due to their awareness of being observed. This is an issue in experiments carried out in a laboratory setting since the laboratory does not resemble the subjects’ normal environment and hence the subjects behave differently, even without noticing this change in behaviors themselves. To alleviate or nullify this change in behavior, researchers have to create experiment setups where the subjects are not or only marginally aware that they are observed. This can be the case with home-based studies where the subjects are observed in their normal day-to-day environment. These home-based studies are enabled by SensorHub.

Through the development of the latest wearables, it is possible to record vital parameters and movement data simultaneously. This poses a chance for advancements in various fields of research, for example, human activity recognition (HAR). Common parameters that can be reliably measured today with portable sensors are e.g., heart rate variability (HRV), blood pressure (BP), body temperature, or skin conductivity (SC). But also continuous measurements such as the recording of an electrocardiogram (ECG) or electroencephalography (EEG) are now possible.

**Figure 1:**
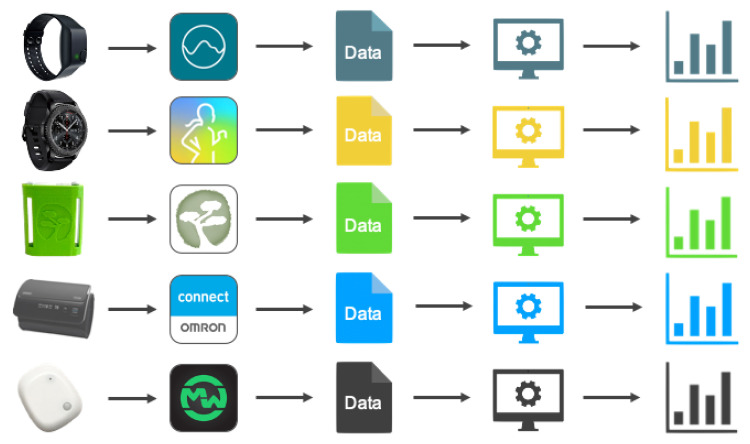
When working with multiple devices, one has to deal with different apps that come with specific manufactures. This often leads to the fact that data is already pre-processed or delivered in different data formats.

Nevertheless, there are use cases that require multiple sensor modalities or sensor locations, for example, when one wants to study the relationship between location, stress, and eating behavior. Here, not only the location and movement of the subject are necessary, but also vital parameters, such as skin conductance. Therefore, usually multiple sensor devices from different manufacturers are needed. This creates a further obstacle, as different device manufacturers work with different applications to evaluate their signals. This in turn leads to an almost insurmountable effort for both the subject and the researcher. Sometimes it is not possible to get the raw data of the sensors but one directly receives pre-processed signals. However, if it is possible to preserve the original data, it is often not possible to compare them with those of other manufacturers because there is no uniform format. This challenge is visualized in Figure 1.

In addition to collecting sensor data for studies, it is important to be able to get point-in-time feedback from the user. Ecological momentary assessments (EMAs) refer to the repeated polling of certain conditions (behavior, feelings, experiences). The idea of EMAs is to bring together the science and practice of clinical psychology by looking at behavior in real-life situations [5]. Taking up the previously described example again, in which eating behavior is to be examined, it is important not to rely solely on pure sensor data, but also to consider other factors. For example, information about emotional states can be collected by means of the questionnaires implemented in SensorHub and the possibility to send them to the user at certain points in time. The final combination of objective measurements and subjective feelings offers enormous potential for studies in everyday life.

In this work, we aim at giving an answer to the research question of how multi-modal and especially multi-sensor data recording can be performed while averting inconsistencies between the sensor data (regarding data format and time offset) and avoiding the effort of coordinating deviating recording procedures among different sensor devices. We also show how we implemented EMAs and how it can collect additional important information.

## 2. Approach

**Figure 2:**
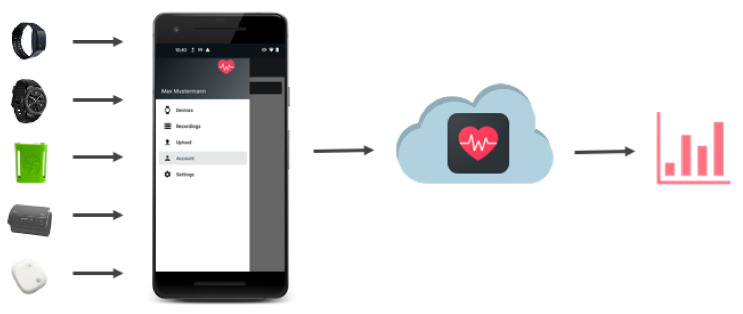
Visualization of our new approach when working with multiple wearables at the same time. The different sensor data is streamed to our SensorHub application using classic Bluetooth or Bluetooth Low Energy (BLE). The collected data can be manually pushed to the server for further research and visualizations.

To overcome the obstacles that come with using many different sensors in home-based studies, we present our application called *SensorHub*. As the name suggests, our application acts as a central component in the communication between several sensors and the subject’s smartphone (cf. Figure 2). SensorHub offers connecting to different wearable devices over classic Bluetooth or Bluetooth Low Energy (BLE) and aggregates the signals in a consistent data format. The data from the sensors is sent to the smartphone in real-time, making it possible to receive immediate feedback. Thus, errors, e.g., in transmission or processing, can be identified directly.

### 2.1. High-Level Architecture

SensorHub consists of multiple components. It offers a frontend in form of an Android application for the subject as well as a dashboard for the researcher. The latter is used to create and manage studies for which sensor data should be collected. Through this dashboard, the researcher can define sensors for a study. Consequently, a potential subject does not need to initialize the sensors for a study, but simply uses the Android app. The defined sensors are shown automatically and can be connected via Bluetooth with one click. By starting a recording, the sensor data is streamed to the app. In the app, there is also the option to provide a questionnaire or let the user record an audio file. Furthermore, uploading the sensor and questionnaire data directly and continuously allows the researcher to monitor the measurements at any time via the dashboard without any further effort from the subject. More detailed descriptions are given in Section 4.1 and Section 4.2, respectively.

### 2.2. Practical Relevance

Inertial measurement units (IMUs), especially accelerometers, seem to be the single most relevant sensor in HAR [6]. However, multimodal sensor fusion, i.e., the use of multiple different sensors such as barometric, thermometric, or even electrocardiographic sensors is also a predominant theme, according to a survey [7]. Especially in energy-consuming activities, ECG is of vital importance [8], which increases its relevance in sports science and medicine. This is supported by another review that states that “Physical health sensors, including heart rate (HR), oxygen saturation (SpO2), BP, ECG, blood glucose (BG), respiratory rate (RR), etc., are sometimes used with motion sensors to detect the activities of subjects for rehabilitation purpose or capturing their vital signals for health condition evaluation.” [9].

Furthermore, the introduction of an individually adaptable questionnaire supports the practical relevance even more. Current research shows that smartphone-based EMA is on the one hand a very active topic and on the other hand offers enormous potential to combine objective measurements and subjective perceptions [10].

This strongly manifests a need for a medical-grade multi-modal sensor and questionnaire data collection tool, which is what we propose in this work.

## 3. Related Work

Multimodal sensing is a challenge in various areas of engineering and research. Hence, there are already solutions in place that provide data collection from different sensors to some extent. However, none of them offers the possibility to perform home-based studies with such little effort on behalf of the subject. For home-based studies, the software system serves as an interface between researcher and subject. The researcher defines what needs to be recorded in terms of sensor selection and placement. The subject performs their normal behaviors, for example at home, and data is collected automatically during this time. Sensor type and placement cannot be accidentally changed by the subject. Data is initially stored on the phone and later transferred to a backend server.

Existing solutions to multimodal sensing can coarsely be subdivided into two categories: Firstly, those that only record data from the internal sensors of the smartphone, such as Phyphox${}^{®}$ (https://phyphox.org/ (accessed on 14 June 2021)) and Physics Toolbox Sensor Suite (https://www.vieyrasoftware.net/ (accessed on 14 June 2021)). And secondly, those that also collect data from external sensors, such as RADAR-base (https://radar-base.org/ (accessed on 14 June 2021)), former Science Journal (https://support.google.com/sciencejournal/answer/9715912 (accessed on 14 June 2021)), and Thryve (https://thryve.health/ (accessed on 14 June 2021)). In the following paragraphs, we briefly describe each of the aforementioned solutions and Table 1 provides a structured comparison.

The open-source smartphone app Phyphox${}^{®}$ has been created at the 2nd Institute of Physics of the RWTH Aachen University in Germany. As the name suggests, the app was developed for physics experiments [11]. Therefore, it focuses mainly on the sensors integrated in smartphones. The possibility to integrate external sensors is currently limited to BLE devices which expose common Generic Attribute Profile (GATT) characteristics. Of the 8 sensor types supported by SensorHub, only 2 use these characteristics, so Phyphox is not a viable alternative. Thus, even though Phyphox provides a backend, it is not suitable for home-based studies due to a lack of compatible external sensors.

Vieyra Software published in March 2017 their Physics Toolbox Suite Pro and their free Physics Toolbox Sensor Suite for Android smartphones. It was developed as a tool for physics experiments on mobile devices for secondary education, universities, and professional settings. Similar to Phyphox, the integration with external sensors is not given in a relevant sense. Furthermore, no backend is provided. Thus, the resulting data cannot be controlled by the researcher during the collection of a home-based study. In contrast, SensorHub provides more external sensors and the possibility to check the collected data while the study is running.

RADAR-base (Remote Assessment of Disease And Relapses) is an open-source platform to collect data from wearable devices. According to their website “the main focus of RADAR-base is a seamless integration of data streams from various wearables to collect sensor data in real-time and store, manage and share the collected data with researchers for retrospective analysis”. In addition to the smartphone sensors, five different wearables (Empatica E4, Pebble 2, Faros, Biovotion, Fitbit) can be integrated into the app. RADAR-base solves a similar problem. It is primarily less user-friendly than SensorHub due to the lack of clear navigation. Furthermore, while SensorHub has an explicit focus on device support, RADAR-base recently even deprecated a device, indicating that this may no longer be a core feature of RADAR-base. It is likely that using RADAR-base with the currently supported devices is still possible, however, an alternative with focus on sensors will help those researchers who cannot use RADAR-base for reasons of device support. RADAR-base additionally offers a so-called questionnaire app. In contrast to RADAR-base, however, the approach of SensorHub is to have all functions integrated in one app in order to keep the additional effort for the subject as low as possible. Therefore, SensorHub offers both sensor data recording and questionnaires in one app. Lastly, RADAR-base does not clearly indicate the state of each sensor, whereas SensorHub provides a graph for each collected data type to ensure the sensors are working properly.

Google’s former Science Journal provided access to the phone’s internal sensors through a user-friendly interface that is directed at students and teachers for use in a classroom setting. Formerly, there was a collaboration with the Arduino Project but accessing external sensors was not a goal of this project. The Science Journal was thus limited with regards to the collection of health data, which SensorHub is specifically focused on.

Thryve caught our attention through a literature search. Developed by mPioneers in Germany in 2017, the application seems to be already established in a productive environment. According to their website “Thryve enables health services to connect all their customers’ device data through one harmonized plug and play API”. With more than 320 wearables being able to integrate, they cover a wide range of available devices. However, since the software is not open-source, details remain hidden. It is not clear whether raw data is available and customization also seems to involve costs. According to their website, Thryve also offers the possibility to provide questionnaires. However, it is still not clear in which form this is possible and to what extent they are adaptable. Furthermore, missing access to the source code poses privacy concerns, since it cannot be certain what happens to the data. Thryve thus does not have value for research into low-level health algorithms. As SensorHub exclusively includes sensors with raw data access, it is much better suited to this use-case.

## 4. Implementation

Our ultimate objective is to perform home-based, observational studies. Therefore, we have to collect multimodal data from various sensors attached to subjects in real-life situations. To achieve this, we utilize various external sensors, smartphones, and a server infrastructure that form a tree-like system architecture.

In effect, our requirements are that the system should be modular, so that support for further sensors is easy to implement. Furthermore, the system should be robust, meaning that common obstacles in data acquisition such as a temporarily unavailable internet connection should not result in data loss. We also emphasize an interface that is easy to understand and use, but without limiting functionality. Finally, we want the system to be open to user input, as users should be able to provide information on what they are currently doing, so data labeling is possible.

In Section 4.1, we describe the used technologies as well as the sensors that are currently integrated into the system and can be used immediately without further customization. In Section 4.2 we explain how the above requirements are satisfied through of architectural and design decisions.

### 4.1. Technologies

In this section, we describe both hardware and software used with and in SensorHub. This includes sensors, transmission protocols, smartphones, databases, as well as tools and software libraries for data visualization and presentation.

#### 4.1.1. Consumer Hardware for Data Acquisition

SensorHub aims to be unobtrusive and avoid frustration or behavioral changes in the subjects. If possible, the sensors used for data acquisition should be small and attached to the subjects comfortably. Hence, we decided to predominantly focus on consumer hardware, since comfort is an important selling point in these products. However, we are not limited to consumer hardware, since all kinds of sensors can potentially be used with SensorHub, as long as these sensors can transfer data either via classic Bluetooth or BLE. In the following, we describe the sensors in use as well as their respective purpose. For a detailed comparison of the IMUs in use see [12].

**Former**  **Biovotion Everion**${}^{®}$
  is a device worn on the upper arm that combines a multitude of sensors. Among the 22 measured parameters are HR, blood oxygen saturation, and skin temperature. See https://support.biofourmis.com/hc/en-us/articles/213108389-Overview-of-the-Everion-device-hardware-components (accessed on 14 June 2021).
**Bonsai**  **Systems${}^{®}$ QuantiMotion**
  is an IMU equipped with a 3D accelerometer and a 3D gyroscope. With dimensions of 36.5 mm × 32.0 mm × 13.5 mm and a weight of 15 g, it is sufficiently small to be strap-mounted at arbitrary positions on the subject.
**Empatica**  **E4**
  is a research-grade device for “real-time physiological data streaming”. The E4 is equipped with a 3-axis accelerometer, an electrodermal activity (EDA) sensor, an infrared (IR) thermopile, and a photoplethysmography (PPG) sensor. It supports on-board data recording as well as data streaming via BLE. See https://www.empatica.com/research/e4/ (accessed on 14 June 2021).
**GaitUp**   **Physilog**${}^{®}$
  is an IMU featuring a 3D accelerometer for measuring linear acceleration, a 3D gyroscope for measuring rotation, and a barometric sensor for measuring pressure (we use the fifth generation Physilog 5). With dimensions of 47.5 mm × 26.5 mm × 10 mm and a weight of 11 g it is sufficiently small to be strap-mounted at arbitrary positions on the subject. See https://research.gaitup.com/physilog/ (accessed on 14 June 2021).
**NeuroSky**  **MindWave Mobile 2**
  is a consumer-grade one-channel EEG device providing a 512 Hz, 12 bit EEG signal as well as derived metrics such as EEG power spectrums. The device is more comfortable than medical-grade EEG devices and effectively limits the required over-the-air bandwidth by recording only one EEG channel instead of the conventional more than 10 channels [13]. See https://store.neurosky.com/pages/mindwave (accessed on 14 June 2021).
**Polar**  **OH1**
  is an optical heart-rate sensor. It can be wrist-worn using an armband or attached to the face using a swimming goggle strap clip. While optical heart rate measurement is not as accurate as electrical heart rate measurement [14], the sensors tend to be more comfortable since no chest strap is required and the position of the sensor can be chosen more freely. See https://www.polar.com/us-en/products/accessories/oh1-optical-heart-rate-sensor (accessed on 14 June 2021).
**Polar**  **H10**
  is a chest-worn electrical heart-rate sensor. The chest strap makes attachment to the subject more complicated but measurements are considered to be more accurate [14]. See https://www.polar.com/us-en/products/accessories/h10_heart_rate_sensor (accessed on 14 June 2021).
**Shimmer3**  **GSR+**
  provides a 3D accelerometer for measuring linear acceleration, a 3D gyroscope for measuring rotation, and a 3D magnetometer for determining its rotational position in space. Furthermore, it can measure EDA and PPG as well as derived metrics such as HR. With this, the Shimmer3 GSR+ adds physiological parameter measurement to a standard IMU. See https://shimmersensing.com/product/shimmer3-gsr-unit/ (accessed on 14 June 2021).
**Bittium**   **Faros 180**
  is a one-channel, waterproof ECG sensor. The Bittium Faros 180 transmits data via classic Bluetooth within a range up to 100 m. It enables ECG sampling up to 1000 Hz and 3D acceleration sampling up to 100 Hz. See https://www.bittium.com/medical/bittium-faros (accessed on 14 June 2021).

#### 4.1.2. Classic Bluetooth and BLE as Data Transmission Standard

The sensors described above transmit data either via classic Bluetooth or BLE. Wherever possible we decided to use BLE as uniform data transmission technology. Just like conventional Bluetooth, BLE operates in the 2.4 GHz ISM band (frequency bands that are reserved for industrial, scientific, and medical purposes and not used for telecommunication purposes), but features significantly lower power consumption (1% to 50% of conventional Bluetooth power consumption, depending on the use case, see https://www.bluetooth.com/learn-about-bluetooth/tech-overview/ (accessed on 14 June 2021)). This is important when using wearables since power supply comes from an integrated battery of limited capacity. Higher power consumption would require larger batteries or more frequent recharging.

#### 4.1.3. Smartphones as Central Unit in Data Aggregation

Nowadays, smartphones are nearly ubiquitously available with about 3.5 billion users worldwide (see https://www.statista.com/statistics/330695/number-of-smartphone-users-worldwide (accessed on 14 June 2021)) and Android is the most prominent mobile operating system with about 85% market share (see https://www.idc.com/promo/smartphone-market-share/os (accessed on 14 June 2021)). Most smartphones support BLE which can be used for communication with the previously describes sensor hardware. On top of that, smartphones offer a decent amount of sensors themselves.

#### 4.1.4. Relational and Time-Series Databases

For persistence and central data storage, we are using PostgreSQL (see https://www.postgresql.org/ (accessed on 14 June 2021)) as relational database management system in conjunction with InfluxDB (see https://www.influxdata.com/ (accessed on 14 June 2021)) as time-series database. PostgreSQL is used for general-purpose data storage since relational databases are easy to use in conjunction with object-oriented programming languages using object-relational mappers (ORMs). However, for performance reasons, we needed a special solution for storing sensor data which have to be stored quickly and are rarely changed. Therefore, we decided to introduce InfluxDB for this very case.

#### 4.1.5. Dashboard for Data Exploration

To make initial data exploration as simple as possible, we introduce a dashboard that presents the measured data. For this, we utilized Flask (see https://palletsprojects.com/p/flask/ (accessed on 14 June 2021)) as web framework and Bokeh (see https://bokeh.org/ (accessed on 14 June 2021)) as visualisation library. The dashboard also provides an interface to define which data should be collected via the app. This includes defining the type of sensor (e.g., Bonsai Systems${}^{®}$ QuantiMotion) and the respective sensor placement (e.g., right wrist).

### 4.2. Main Components

**Figure 3:**
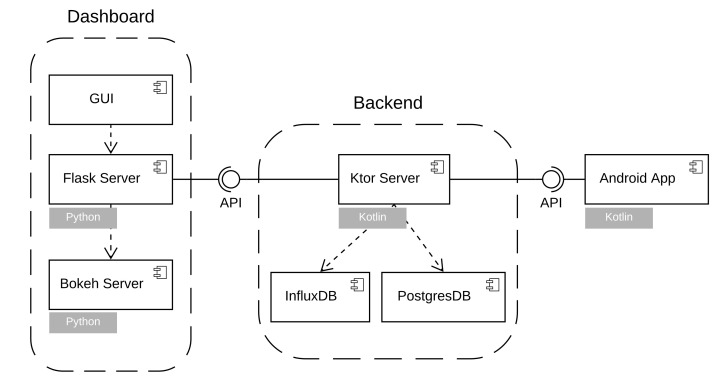
Component diagram showing the three main components of SensorHub: App, Backend, and Dashboard. Dashed arrows indicate usage.

SensorHub consists of three main components: Smartphone app, backend, and dashboard. These components were developed using agile software development methods, specifically extreme programming (XP) as developed by Kent Beck [15]. The component diagram in Figure 3 shows the interaction and technologies of the SensorHub system. The three components and their respective purpose will be described in the following subsections.

#### 4.2.1. Smartphone App

The SensorHub app is written in Kotlin (see https://kotlinlang.org/ (accessed on 14 June 2021)) and running on Android smartphones (versions from Android 6.0 Marshmallow (API 23) are supported). The app is able to collect data from various sensor devices (cf. Section 4.1) as well as internal sensors of the smartphone. For data collection from sensor devices, Bluetooth connections to supported sensors are established and subsequently used for data transmissions from the sensor devices to the app.

SensorHub offers the additional option to include EMA in form of questionnaires in a study. These include both textual prompts and audio recordings. In order to be well-prepared for different research questions and thus studies, we implemented Likert scales as well as affective sliders [16] for the questionnaires. It can be configured individually if and when a reminder should be sent to the subject.

**Figure 4:**
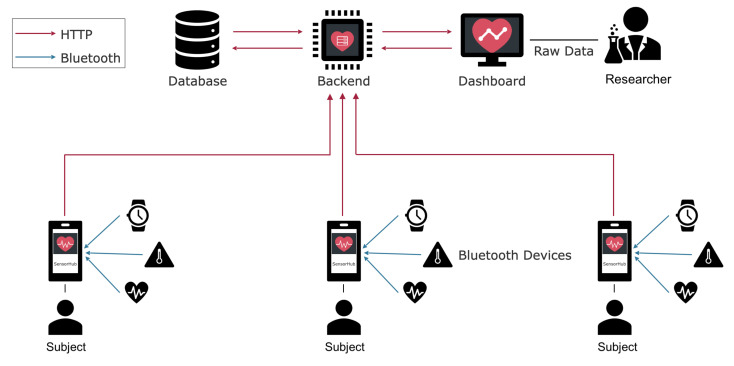
Overview of the communication happening within SensorHub. Sensor devices communicate with the app on the smartphone via Bluetooth. Every further communication happens via HTTP(S).

The illustration in Figure 4 provides a clear overview of the individual roles, devices, and the communication between the app operated by the subject and the dashboard operated by the researcher. Internal smartphone sensors can be accessed right away via Kotlin.

The app is also responsible for initial data storage. Measured and received data are kept on the smartphone until they can be transmitted to the backend component.

Furthermore, the app provides a tagging interface to label the data. The tags displayed in the app were defined by the researcher via the dashboard for the respective study and cannot be changed in the app. There are two types of tags: Single events and start-stop events. Single events denote a specific point in time where some event happened, for example, medication intake. Start-stop events denote a period of time, for example over a performed exercise. The subject is responsible for selecting and deselecting the tags during a recording. Tags are stored along with sensor data and, in the end, also transmitted to the backend.

To ease the process of adding new sensors, the app features a modular architecture. All modules for communication with sensor devices are subclasses of a *Device* superclass. This simplifies adding new devices and ensures a consistent interface for every device. Each module is contained in a dynamic delivery module, allowing the app to automatically load only modules required for a study from the Google Play Store (see https://play.google.com/ (accessed on 14 June 2021)). This lessens the load a study imposes on the user’s phone storage.

#### 4.2.2. Backend

The SensorHub Backend works as an intermediary between app and dashboard and is responsible for persisting collected data. Architecturally, the backend consists of three Docker (see https://www.docker.com/ (accessed on 14 June 2021)) containers:

1. A container running a Ktor web appliance (see https://ktor.io/ (accessed on 14 June 2021)) exposing a REST API for storing and retrieving data.
2. A container running an InfluxDB time-series database which is used by container 1 for storing sensor data (which are time-series).
3. A container running a PostgreSQL for non-time-series data, such as metadata on subjects and study configurations. This container is also used by container 1.

We decided to use two different database management systems because performance characteristics for the different data types in question are vastly different. Hence, two tailored database solutions provide enhanced performance as compared to one general-purpose solution. Sensor data are stored in InfluxDB, a times series database that can readily handle large consecutive insert operations. Postgres is used for less data-intensive use cases, such as user management and study meta configuration.

#### 4.2.3. Dashboard

The SensorHub dashboard offers easy access to the data provided by the backend. This includes time-series and non-time-series data (cf. Section 4.2.2). Furthermore, it is possible to configure studies and visualize the uploaded data. The dashboard is split up into two main components:

1. A Bokeh server for plotting data. From there, time-series data are requested from the backend. Generated plots are then used by the following component 2.
2. A Flask web server that is responsible for everything but plotting data. This includes handling requests to the backend, study configuration, and user management. Plots are requested from component 1 via a WebSocket connection (cf. [17]).

## 5. User Interfaces

As described in the previous section SensorHub offers two user interfaces: the dashboard for the researcher and the smartphone app for the subject. The interfaces were designed to be as self-explanatory and clear as possible.

### 5.1. Dashboard Interface

The dashboard provides four areas. In the *Sensor Data* section, the raw data can be filtered and downloaded. In addition, simple plots can already be created, which help the researcher understand the data immediately.

In the *Study* area, new studies can be created and new subjects can be defined for them. Subject accounts are only identifiable via a pseudonym that is not associated with any personal information through the SensorHub infrastructure. Hence, subject accounts are anonymous as far as SensorHub is concerned. SensorHub is thus not responsible for the data storage of subjects’ personal data. Accordingly, the anonymous accounts for the subjects must be submitted by the researcher themselves so that the subject can log into the Android app and collect data for a study.

**Figure 5:**
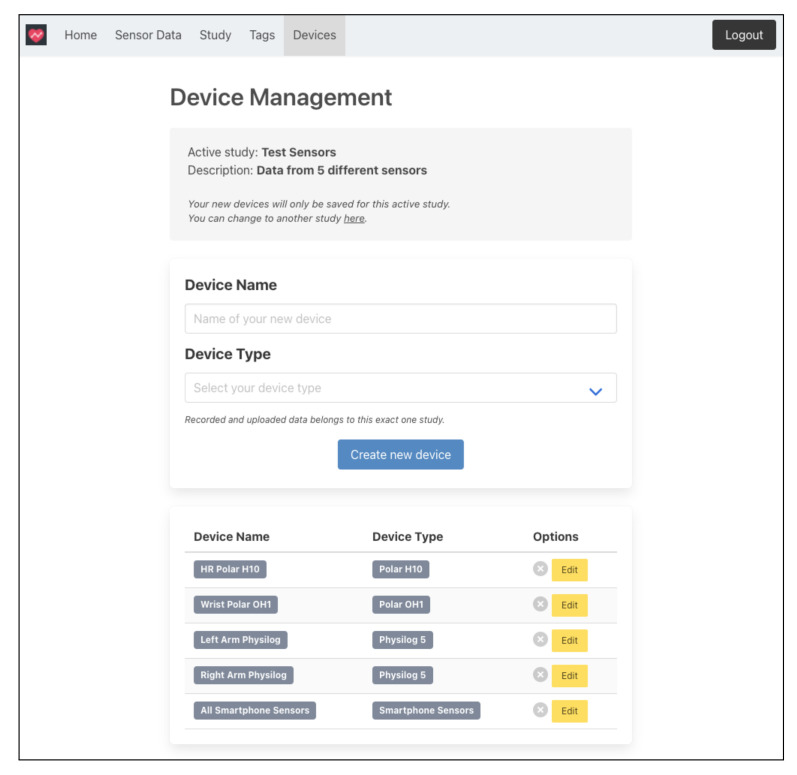
Device management page from SensorHub dashboard.

The *Tags* and *Devices* sections always refer to a specific study. In the *Tags* section, these can be defined. These tags are then available to the subject in the app so that data can already be labeled during data recording (cf. Section 4.2.1). Figure 5 shows the device management page for a study. In the *Devices* section, any number of devices can be defined for a study. In the *Device Type* drop-down menu, the researcher can choose from all the wearable types that have been implemented so far. The devices already created for the study are listed at the bottom of the page.

### 5.2. Android Smartphone App Interface

The scope of the smartphone app is also limited to the essential functions so that it can be operated by any subject without much prior knowledge and with little effort. Once a user logs in with their assigned account, the configuration for their study is automatically downloaded.

**Figure 6:**
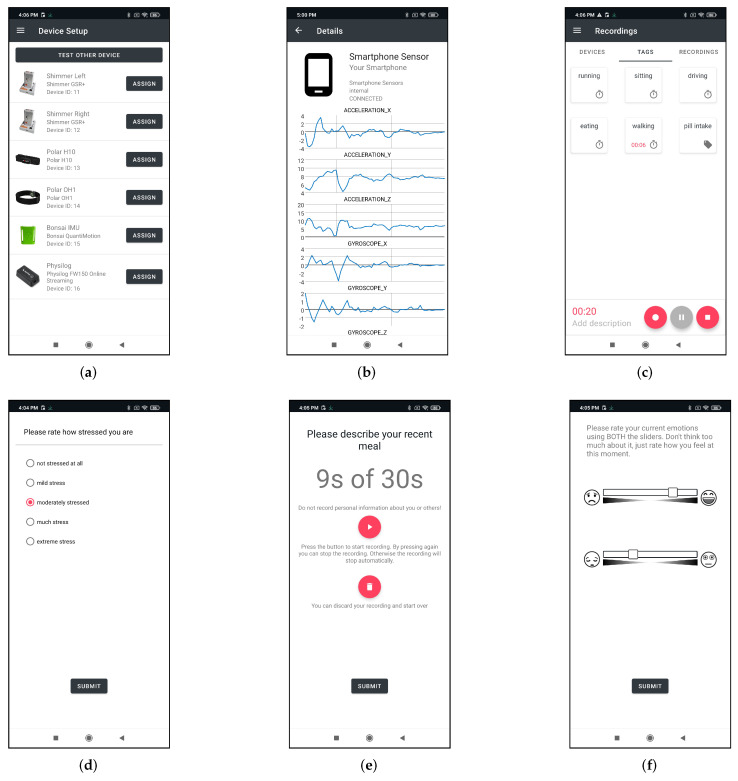
Different views from the SensorHub Android application. (**a**) *Device Setup* screen showing the defined devices for a study, (**b**) Streaming data visualization of the sensors from the smartphone, (**c**) *Recordings* screen with six predefined tags where one is active, (**d**) Exemplary questionnaire with a Likert scale, (**e**) Example for an audio recording questionnaire, (**f**) Sample questionnaire screen using affective sliders.

All the user has to do is to pair the devices with the app (see Figure 6a for an example). All wearables to be paired are listed under the screen *Device Setup* and can be connected easily. To check whether the wearables are sending data, the user can click on the respective device and get a live overview in the form of plots of the respective sensors (see Figure 6b). The data recording can then be started in the *Recordings* area. The tags defined by the researcher are available to the user here. As can be seen in Figure 6c, after data acquisition is started, one can select and deselect one or more tags, depending on which activity is currently being performed.

Furthermore, it is possible to define EMAs for studies. These can contain different forms of questionnaires. Possible formats are questionnaires with Likert Scales (see Figure 6d), audio files can be recorded (see Figure 6e) or affective sliders can be defined for a survey (see Figure 6f). A reminder to fill out the questionnaires can be predefined so that the user is reminded to provide the information at certain times during the day.

In the *Upload* section, all completed data recordings are available. These can be uploaded independently by the user. However, since these raw data files are usually very large, an unlimited internet connection is necessary for the upload.

SensorHub is still being developed and improved. Feedback from users is collected and included in further development.

## 6. Evaluation and Discussion

Building SensorHub showed that recording data from different sensors at the same time entails some challenges. Some of them are imposed by the technologies (e.g., hardware, transmission protocols) in use and cannot be affected by us in a meaningful way. These challenges will be discussed in this section as *limitations*. However, some other challenges are based on SensorHub’s design and hence are under our control. These will be discussed as *future work*. However, we first want to discuss potential use cases and explore general themes on how to use the data generated by SensorHub.

### 6.1. Potential Use Cases

SensorHub is a tool for data collection and as such mostly use-case agnostic. The only prerequisite being that there needs to be an implemented sensor module for each type of sensor in use. On that condition, all kinds of sensor data can be collected with the SensorHub app and then downloaded via the SensorHub dashboard. This can be used for studies in biomechanics, HAR, biomedical engineering, and many more areas. The collected data are provided as tabular data files (comma-separated values (CSV), Apache Parquet) and can be processed in any kind of way that suits the research question at hand. Examples of means of data processing are statistical analysis, visualizations, and training of machine learning models. Essentially, SensorHub is a tool that is agnostic to both the field of research it is used in and the subsequently applied data processing steps, which depend on the research question at hand.

In the following, we present two projects that we have carried out with SensorHub. We present the role of the software and show the need for a multimodal sensor data collection tool based on the results. Detailed information on the projects can be found in the respective publications.

#### 6.1.1. Project Exercise Exertion Prediction

This subsection describes the use of SensorHub in a project to predict perceived exertion during resistance training to avoid injuries resulting from overtraining [18]. To this end, IMU and ECG data from 16 participants performing squats on a flyhweel training machine was collected with SensorHub. In total six IMUs (Physilog 5) and one ECG sensor (Faros 180) streamed their data in real-time to the SensorHub application installed on an Android smartphone. The IMU sensors were placed on the right and left thigh as well as the right and left calf, one IMU was placed on the back and another one was placed on the chest. Furthermore, the subjects were repeatedly asked about their subjective exhaustion using Rating of Perceived Exertion (RPE) measured by means of a Borg scale. This scale is a numerical scale that ranges from 6 to 20, with 6 meaning *no exertion at all* and 20 meaning *maximal exertion*. The data was analyzed using multiple regressors, such as Support Vector Machines (SVMs), Random Forests, and Gradient Boosting Regression Trees. Detailed information about the study can be found in the corresponding paper. Regardless of the performance of individual models, the results improved significantly when combining the movement data (collected with IMU sensors) and the HRV (collected with an ECG sensor), instead of using solely IMU data. The results in terms of the coefficient of determination ($R^{2}$) and mean squared error (MSE) are shown in Table 2. $R^{2}$ stands for the proportion of deviation from the target value that a model reproduced. For a perfect prediction $R^{2}=1$, for a mean prediction $R^{2}=0$, and for $R^{2}<0$ the prediction is worse. Ultimately, the study shows that a multimodel sensor data collection tool, like SensorHub, not only facilitates data acquisition and processing, but also contributes to better results.

#### 6.1.2. Project *EatMaps* Using EMAs

In the following, we will present the use of SensorHub in our project *EatMaps* and explain why SensorHub offers significant added value for projects of this kind.

The *EatMaps* project is funded by the Federal Ministry for Economic Affairs and Energy, Germany. In this project, we want to observe how affective states and user contexts correlate with eating habits in everyday life. The motivation for this study comes from the increasing number of diabetic patients. The global prevalence of diabetes among adults above 18 years has risen from 4.7% in 1980 to 8.5% in 2014 [19], with obesity as the leading risk factor for type 2 diabetes. In addition, stress is suspected of promoting excessive food intake and weight gain [20]. Moreover, affective states such as anxiety, anger, depressed mood, and other negative emotions also correlate with eating behavior [21]. We are conducting this study along with the Oviva AG (https://oviva.com/de/de/ (accessed on 14 June 2021)), which is our industrial partner for this third-party funded project. Oviva has several years of expertise in of monitoring the eating habits of diabetic and obese patients. They provide and support digital solutions for patient-oriented therapy to change dietary behavior via a mobile application.

In order to investigate this multitude of influencing factors in more detail, different devices and signals are required. We collected different types of data, including physiological and movement signals, internal sensor data from the smartphone, and additional information in form of EMAs. So far, in a study, we have recorded data from 11 subjects over an extended period of time with at least 8 h per day.

To collect physiological and motion signals, and to identify the eating activities, we used the Shimmer3 GSR+ devices (for more information please refer to Section 4.1.1) with a sampling frequency between 50–52 Hz. The following signals and values were recorded: PPG, EDA, temperature, pressure, and IMU data. We used SensorHub’s questionnaire feature to collect EMA data and additional internal sensor data, as e.g., IMU data, Global Positioning System (GPS), light and Bluetooth data which are used to understand the social interaction. The EMA data included questionnaires that are related to stress, affective states, wake-up time, social interaction, background noise recordings, and voice prompts about users’ moods or feelings. The EMAs were polled every 2 h from 9:00 a.m. to 9:00 p.m. The subjects were also asked to take images of the food they eat when they are using the sensors.

**Figure 7:**
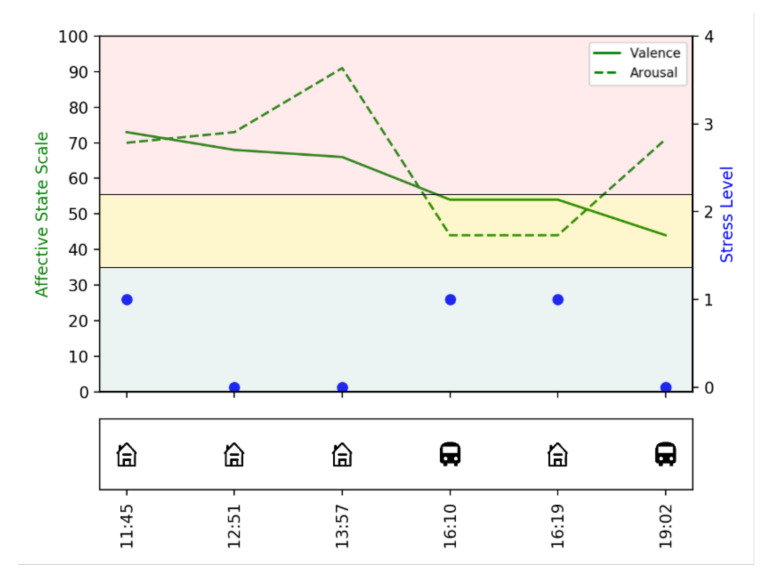
Example visualization of the EMA and GPS data from one subject and one day.

With the help of EMAs and GPS data, we can gain insight into a person’s emotional state and analyze whether it changes based on location, for example. Therefore, we asked for the arousal and valence (on a scale from 0 to 100 [16]) and the stress level (from 0 meaning *not stressed at all* to 4 meaning *very stressed*). Specific locations, as e.g., *at home* or *in public transport*, were derived by using the GPS signal. An example visualization of one subject’s data from one day can be found in Figure 7. Note that the three-part division of the affective state scale means *low* between 0 and 45, *neutral* between 45 and 55 and *high* from 55. Here, for example, it can be seen that for this subject the stress level was always very low, even when the subject was very aroused.

The big advantage of using SensorHub in this study was having all the necessary functions integrated into one application. The subjects could fill out the questionnaires via SensorHub as well as connect the sensors and have data recorded. In addition, the internal sensors of the smartphone could be used.

### 6.2. Technical Evaluation

To evaluate whether SensorHub’s backend is capable of handling incoming data with sufficient speed, we performed a data ingestion benchmark on the backend as a stress test. The SensorHub backend is designed to ingest sensor data from multiple patients faster than the sampling rate to allow sporadic upload of data cached on the smartphone. To verify that this requirement has been fulfilled, we conducted benchmarks with the open-source WRK tool (see https://github.com/wg/wrk (accessed on 14 June 2021)), that allows to specify a duration as well as the number of threads creating a specific number of connections. To mimic a connection with the app’s upload module, batches of 3000 random sensor data were created. Such batches were continuously uploaded for a duration of 10 min in 8 threads on a single machine utilizing 64 connections overall. As this creates mainly network load for the machine running WRK, that in turn mostly needs to wait for responses, this is not limited by the requesting machine’s CPU. The virtual machine (VM) running the backend for these tests had 3 virtual cores and 3GB of RAM. During the entire test run, the 3 cores were loaded on average to 49%±21.47% with a minimum of 3% and a maximum of 93%. Once the Java virtual machine (JVM) completed its warm-up phase, the memory load of the entire system did not exceed 2 GB. This includes the PostgreSQL DB and the InfluxDB storing the data on disk. The average network ingest speed was 12.7 MB/s ± 1.08 MB/s, while the InfluxDB had to write on average 3.4MB/s±2.7MB/s to disk, suggesting a large compression factor with regards to the JSON format that the backend accepts.

Ultimately, 12,500 requests were made with an average latency of 3s±0.793s. This benchmark proves that studies producing samples at a rate of up to 850 Hz (e.g., using an ECG sensor with 500 Hz that also records 3 IMU axes at 100 Hz plus 50 anomaly tags per second) are feasible for up to 8 people who have been unable to upload for an hour and are now attempting to upload at exactly the same time (as 12,500 requests of 3000 sensor data equal 37,500,000 data points). During normal usage, the upload is synchronized, so the benchmark represents a worst-case scenario.

### 6.3. Limitations

The technologies in use, i.e., consumer sensing hardware, mobile phones, and the Bluetooth transmission standard, expose SensorHub to some limitations. Most notable are quantitative limits like a maximum number of concurrently connected sensors, limited data rates, and packet loss, as well as battery drainage. Nevertheless, the first measurements show that by using BLE a study over a longer period of time (~1 h) is possible even with many devices (5 IMU sensors) and high frequency (512 Hz) without significant battery losses (less than 3%). But there are also qualitative limitations, as we were not able to access raw data with some devices. These limitations are discussed in the following.

#### 6.3.1. Data Rate Limitations and Packet Loss

While BLE theoretically features data rates of up to 2 Mbit/s, practical application throughput is much lower, i.e., 0.27–1.37 Mbit/s (https://www.novelbits.io/bluetooth-5-speed-maximum-throughput/ (accessed on 14 June 2021)). This causes issues when larger amounts of data are to be transferred, as it is for example the case with EEG signals. Even with a smaller set-up of 16 electrodes, 125 Hz sampling frequency, and 32 bit per sample, an EEG produces $16\cdot 125\;s^{-1}\cdot 32\;\mathrm{bit}=64\;\mathrm{kbit}/s$ which might exceed the capabilities of certain BLE chipsets in some cases, especially when not only EEG is recorded but also data from other sensors at the same time.

In addition to data rate limitations, we also experienced packet loss when multiple sensors were connected at the same time. For instance, in the data received by phone, the sampling frequency appeared to drop occasionally in a manner as if divided by small integers ($f_{s}^{\prime }=f_{s}/i$ for i=2,3,…). Our investigation shows that data loss increases when the number of concurrently connected sensors increases. Hence, we hypothesize that data is lost whenever two or more data packets arrive at the same time because the phone can only handle one data packet at a time.

However, both data rate limitations and packet loss seem to be primarily a theoretical problem with only minor practical relevance. This is shown by two evaluation studies we performed using a Pixel 4a (Google, California) phone. Firstly, in a study with 21 participants performing one session each that was conducted with SensorHub, a 1000 Hz ECG was recorded together with its 100 Hz 3-axis accelerometer without any connection or data loss. Secondly, during another set of experiments, we were consistently able to record 6 IMUs with a combined data rate of 2304 Hz, i.e., 384 Hz per IMU, without any data loss.

#### 6.3.2. Upper Limits in Number of Sensors

There are limitations in software and hardware on the maximum number of simultaneous connections an Android phone can have. For one, the oldest Android version supported by our App, i.e., Android 6, has a limit of 7 concurrently connected BLE devices (see https://android.googlesource.com/platform/external/bluetooth/bluedroid/+/master/include/bt_target.h#1428 (accessed on 14 June 2021) and https://android.googlesource.com/platform/system/bt/+/android-6.0.1_r81/bta/gatt/bta_gattc_int.h (accessed on 14 June 2021)). However, as this is not a publicly specified value, a specific study must verify the number of devices supported by the handset to be used. While no documents exist to show the number of devices supported by specific smartphones, many experience reports can be found (see https://stackoverflow.com/a/41367864, https://stackoverflow.com/a/40890325, and https://stackoverflow.com/a/37756809 (accessed on 14 June 2021)).

Hardware limitations can also be introduced due to sharing the same antenna for Wi-Fi and Bluetooth (see https://interrupt.memfault.com/blog/ble-throughput-primer#coexistence (accessed on 14 June 2021)).

#### 6.3.3. Missing Raw Data Access

With many consumer-grade wearable sensors, especially smartwatches, we were unable to determine whether access to raw sensor data (e.g., the PPG signal) is provided or only preprocessed data (e.g., pulse frequency) are available. Since we do not want to rely on proprietary preprocessing algorithms, SensorHub currently supports only a selection of sensors where raw data access could be confidently assumed beforehand (see Section 4.1).

### 6.4. Future Work

As common in software development, SensorHub also faced the trade-off of functionality vs. simplicity [22]. We faced the design decision of whether we wanted to implement SensorHub in the style of a minimal viable product as a tool for recording sensor data with a phone and nothing more (no backend, no dashboard) or if we wanted to have a platform approach that is implementation-wise more complicated but also offers more functionality for researchers. Nevertheless, with additional functionality comes additional complexity. This needs to be reduced or hidden externally in order to achieve user-friendly interfaces for all users of the system (not only subjects, but also researchers and system administrators). We hence plan to further improve SensorHub through multiple iterations of user studies.

Furthermore, there will always be new devices that could be interesting for studies and thus should be implemented in SensorHub. While many different sensors for different modalities are already implemented, there are constantly new sensor requirements that come up while using SensorHub. Hence, a substantial amount of future work will be the implementation of more sensor types to be used with SensorHub.

## Figures and Tables

**Table 1:** A structured comparison of related work with SensorHub shows the unique advantages of SensorHub over alternative solutions. Cells with a ’?’ mark information that is not included in public information about the respective system.

	Open Source	External Sensors	EMA	Home- Based ^1^ Studies	Visuali- Zations	Backend ^2^
Phyphox	GNU GPL	no	no	no	yes	yes
Physics Toolbox Suite Pro	no	no	no	no	yes	no
RADAR-base	Apache-2.0	yes (5) ^3^	yes ^4^	no	yes	yes
Science Journal	Apache-2.0	yes ^5^	no	no	yes	yes ^6^
Thryve	no	yes	yes	?	?	?
*SensorHub*	MIT	yes (8) ^7^	yes	yes	yes	yes

^1^ Using the system for study execution is possible without frequent contact between researcher and subject. ^2^ Data is stored not only on the phone but there is also a backend server able to handle large amounts of data and store them securely. ^3^ Empatica E4, Pebble 2, Bittium Faros, Biovotion Everion, Fitbit Charge 2. ^4^ In form of an additional application. ^5^ Can connect to Arduino and Vernier devices. ^6^ Via Google Account. ^7^ See paragraph Consumer Hardware for Data Acquisition in Section 4.1.

**Table 2:** Results for the models Gradient Boosting Regression Tree (GBRT), SVM with Linear (SVRL) and Radial Basis Function (SVRR), and Random Forest (RF) for using either solely IMU data or a combination of IMU and HRV data.

	$R^{2}$		MSE	
Model	IMU	IMU + HRV	IMU	IMU + HRV
GBRT	−0.01 ± 0.12	0.48 ± 0.30	2.14 ± 0.49	1.45 ± 0.44
SRVL	−0.03 ± 0.13	0.40 ± 0.18	2.19 ± 0.59	1.71 ± 0.67
SVRR	−0.05 ± 0.26	0.22 ± 0.19	2.24 ± 0.81	1.89 ± 0.49
RF	0.08 ± 0.10	0.52 ± 0.06	2.09 ± 0.64	1.51 ± 0.45

## Data Availability

The study did not report any data.
